# Ultrasound versus angiographic guided access in transfemoral TAVI: intra-operator evaluation of vascular and bleeding complications

**DOI:** 10.1007/s00392-025-02805-2

**Published:** 2025-12-15

**Authors:** Niklas Lankisch, Gianmarco Iannopollo, Oliver Dumpies, Ahmed Abdelhafez, Johannes Rotta detto Loria, Ines Richter, Hans-Josef Feistritzer, Steffen Desch, Thilo Noack, Holger Thiele, Nicolas Majunke, Mohamed Abdel-Wahab

**Affiliations:** 1 Department of Internal Medicine/Cardiology and Department of Structural Heart Disease/Cardiology, Heart Center Leipzig at Leipzig University, Strümpellstr. 39, 04289 Leipzig, Germany; 2 Department of Cardiology, Ospedale Maggiore Carlo Alberto Pizzardi, Bologna, Italy; 3 Department of Structural Heart Disease/Cardiac Surgery, Heart Center Leipzig at Leipzig University, Leipzig, Germany

**Keywords:** TAVI, Vascular access, Ultrasound, Angiography, Roadmap

## Abstract

**Background:**

Both angiography roadmap (RM) and ultrasound (US) are commonly used to obtain femoral arterial access during transfemoral transcatheter aortic valve implantation (TAVI). In this analysis, we sought to evaluate the effect of implementation of an US-guided approach on vascular and bleeding complications.

**Methods:**

Vascular complications and bleeding at the main access site were compared using 4-year data from two experienced TAVI-operators, who changed their practice from an exclusively RM- to an exclusively US-guided technique for access in transfemoral TAVI.

**Results:**

A total of 1026 patients were analyzed (RM: n = 485, US: n = 541) with a mean age of 80.7 ± 6.3 years; 47.7% were female and 37.1% received a balloon-expandable valve. Main access vascular complications, bleedings, or their composite were lower in the US-group (RM vs. US: 16.1% vs. 8.3%, p < 0.001). US was a protective factor for vascular complications, bleeding and their composite (adjusted odds ratio [OR] 0.51, 95%-confidence interval [CI] 0.33–0.77, p = 0.002; adjusted OR 0.46, 95%-CI 0.28–0.78, p = 0.003; and adjusted OR: 0.47; 95% CI 0.32–0.70, p < 0.001, respectively). Fluoroscopy time (14 [interquartile range (IQR) 11 – 20] min vs. 13 [IQR 10 – 17] min, p < 0.001), contrast use (88 [IQR 69 – 111] ml vs. 84 [IQR 65 – 110] ml, p = 0.049) and procedure time (52 [IQR 44 – 67] min vs. 49 [IQR 41 – 62] min, p = 0.02) were lower in the US-group.

**Conclusions:**

US-guided femoral access was associated with significantly fewer complications compared with RM-guidance, supporting its adoption even among operators experienced with angiographic guidance.

**Graphical Abstract:**

Central illustration

(Top) Visual representation of the design of the study with examples of the used techniques: (Top left) RM and (Top right) US. (Bottom) Rates of the combined primary endpoint of vascular complications and bleedings at the main access site (middle), main access vascular complications (left) and procedural characteristics (right).

RM = Angiographic Roadmap; US = Ultrasound, VARC-3 = Valve Academic Research Consortium 3

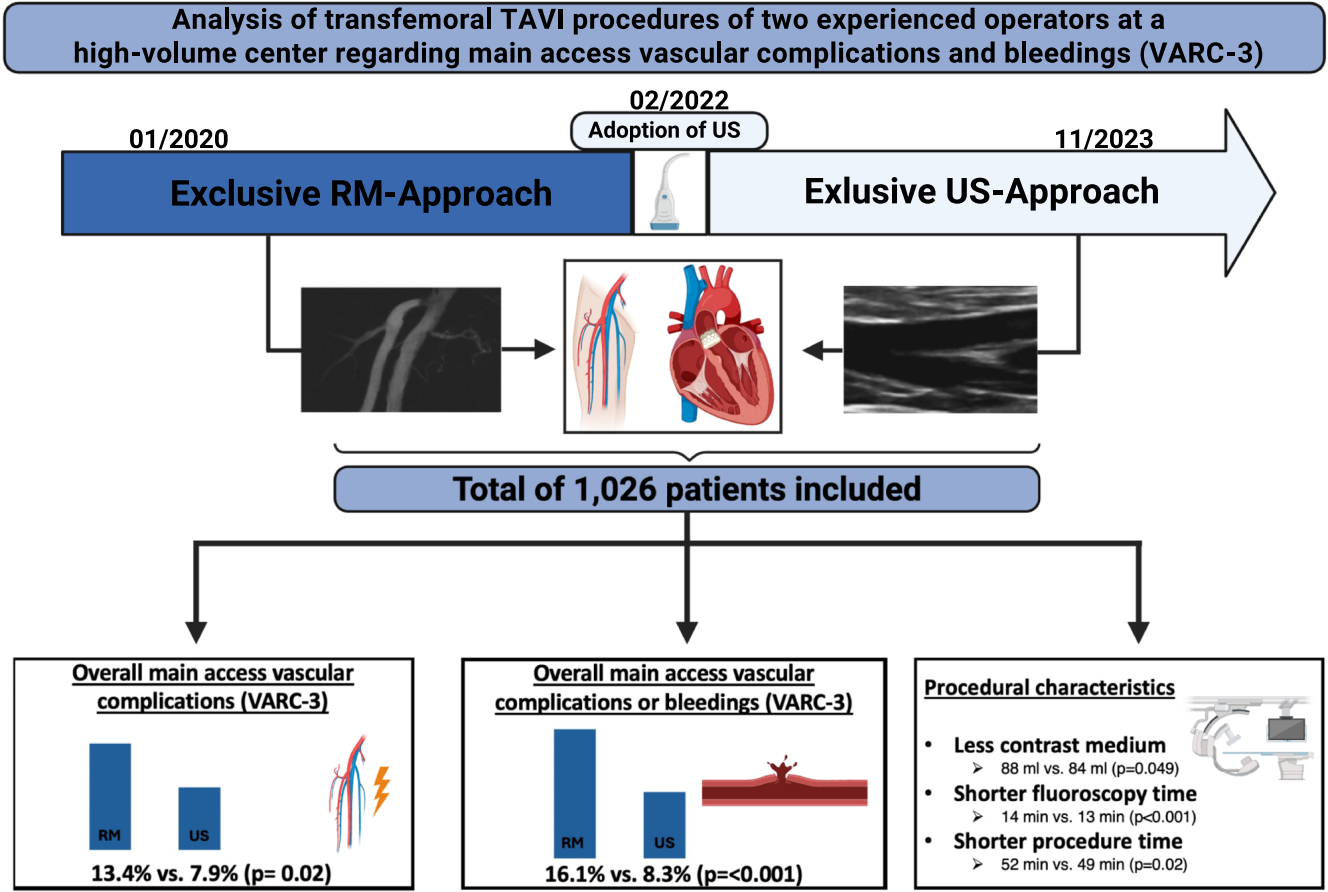

**Supplementary Information:**

The online version contains supplementary material available at 10.1007/s00392-025-02805-2.

## Introduction

Vascular and bleeding complications remain common during transfemoral transcatheter aortic valve implantation (TAVI) procedures, imposing a significant burden on affected patients [1–5].

The convincing results of recently published randomized trials in low- and intermediate-risk patients with severe symptomatic aortic stenosis, along with the expected demographic transition in many Western countries are leading to an increasing population of patients requiring treatment in the near future. These developments highlight the relevance of further improving the safety and efficacy of TAVI procedures [6–10].

To minimize access-site complications, it is crucial to perform a precise puncture of the common femoral artery (CFA), ideally about one centimeter proximal to the femoral bifurcation. To achieve this, angiography “roadmap” (RM)-guided and ultrasound (US)-guided percutaneous puncture techniques are most commonly used to obtain arterial access during transfemoral TAVI, since the anatomical landmark technique does not provide comparable precision [11].

Unlike percutaneous coronary interventions (PCI) [11–13], randomized comparisons of puncture techniques evaluating procedures requiring large-bore arterial access are lacking. A pooled meta-analysis of observational studies suggested a clear advantage for US-guided access for transfemoral TAVI [14]. This is further supported by the findings of a recent German observational study, where the use of ultrasound was associated with less vascular and bleeding complications [15].

However, operator experience and familiarity with angiographic guidance, together with the lack of evidence from randomized trials, appear to prevent adoption of US-guidance in some institutions. In the recently published ACCESS-TAVI trial [16], which included contemporary TAVI procedures from four German centers, US-guidance was performed in less than 60% of all procedures. Similar or even lower rates have been recorded in several national registries [14, 15, 17]. This may be partially explained by an expected little additional value of a different technique on individual complication rates of experienced operators. Therefore, the present study aimed to assess rates of access-related vascular and bleeding complications of experienced TAVI operators, who changed their practice from an exclusively angiographic to an exclusively US-guided technique for femoral puncture during transfemoral TAVI.

## Methods

### Patient population

This study is a retrospective analysis of a prospectively conducted institutional registry at the Heart Center Leipzig at Leipzig University. It included all transfemoral TAVI procedures performed between January 2020 and November 2023 by two high-volume, experienced operators (NM and MAW) with a cumulative volume of 2513 TAVI-procedures prior to the beginning of the analysis. Both operators had fully adopted an US-guided approach for main access after previously relying exclusively on RM-guidance. They transitioned to US guidance in February 2022 and March 2022, respectively. Notably, other procedure related factors were not altered during the period of the analysis. Data were collected after patients provided informed consent at the time of their index procedure, in accordance with the local ethics committee approval and the Declaration of Helsinki (Leipzig TAVI registry, clinicaltrials.gov identifier NCT0501545).

All patients were evaluated by a multidisciplinary heart team before the procedure, and computed tomography angiography (CTA), including assessment of the femoral arteries, was performed to determine the most suitable access route for each patient. Patients who underwent additional procedures (such as leaflet modification), emergency interventions or required surgical cut-down were excluded from the analysis.

### Procedural details

If access was obtained using the RM-approach, contrast medium was injected through a 6 F pigtail catheter advanced from the contralateral accessory femoral or radial access in most cases. After contrast administration, the CFA and its bifurcation were visualized angiographically. A “roadmap” image was then created by subtracting and overlaying the angiogram onto a static image of the iliofemoral vessels to guide CFA puncture in real time.

For US-guided access, a linear ultrasound transducer was used to visualize the CFA, its bifurcation, and the femoral vein in both short- and long-axis views. In short-axis, the transducer was moved to identify the bifurcation and to select a puncture site on the CFA free of anterior wall calcification. Under real-time ultrasound guidance, the needle was then advanced through the skin until it entered the CFA, ideally on the anterior wall at the 12 o’clock position.

### Study endpoints

The primary clinical endpoint was a composite of vascular complications and bleeding events at the main access site, both defined according to Valve Academic Research Consortium-3 (VARC-3) criteria [18]. Secondary endpoints included major and minor vascular complications at the main access site, type of bleeding events (type 1–4 per VARC-3), acute kidney injury, and procedural metrics such as procedure time, contrast volume, and fluoroscopy time.

### Statistical analysis

Baseline characteristics of both groups, including demographic, clinical, echocardiographic and procedure-related factors were compared using a Student’s *t*-test or Mann–Whitney U test for continuous variables, depending on variable distribution. Binary variables were compared with a Pearson Chi-square test.

Multivariable logistic regression analysis was performed to identify predictors of the primary endpoint and to evaluate the independent relationship between the puncture strategy and the primary outcome. Models were adjusted for clinically relevant covariates as well as variables with a p-value < 0.5 in univariable analysis. A two-sided p-value < 0.05 was considered statistically significant. All statistical analyses were performed using SPSS software, version 26.0 (IBM Corp., New York, USA).

## Results

### Baseline characteristics

A total of 1026 patients who underwent transfemoral TAVI by the two operators between January 2020 and November 2023, were included in the analysis. Overall, 485 patients underwent TAVI using the RM-approach while 541 patients were included in the US group. Except for slightly higher rates of diabetes mellitus, hypertension, and direct oral anticoagulant use in the US group, both cohorts were otherwise well balanced at baseline* (*Table 1*).*

**Table 1:** Baseline clinical characteristics

	**RM (n = 485)**	**US (n = 541)**	*p-value*
Age (years)	80.4 (± 6.4)	81.1 (± 6.1)	*0.07*
Female (%)	235 (49%)	254 (47%)	*0.63*
BMI (kg/m^2^)	28 (± 5.3)	27.9 (± 5.3)	*0.84*
STS-score	3.4 (2.4–5.2)	3.7 (2.4–5.6)	*0.05*
NYHA > II (%)	325 (67%)	373 (69%)	*0.51*
**Comorbidities**			
Hypertension	456 (94%)	525 (97%)	**0.01**
Diabetes mellitus	175 (36%)	243 (45%)	**0.01**
– Insulin-dependent	73 (15%)	92 (17%)	*0.39*
CKD stage 3*	286 (59%)	292 (54%)	*0.11*
PAD	39 (8%)	49 (9%)	*0.56*
CAD	247 (51%)	292 (54%)	*0.20*
– Prior MI	68 (14%)	60 (11%)	*0.16*
Prior stroke	53 (11%)	70 (13%)	*0.32*
**Prior cardiac surgery/intervention**			
CABG	29 (6%)	22 (4%)	*0.16*
SAVR	24 (5%)	22 (4%)	*0.50*
TAVI	3 (0.6%)	11 (2%)	*0.05*
TEER-mitral valve/tricuspid valve	1 (0.2%)	0	–-
Valvuloplasty of the aortic valve	4 (0.8%)	4 (0.7%)	*0.88*
Other prior valve surgery	12 (2.5%)	2 (0.4%)	**0.004**
**Echocardiography**			
Left ventricular ejection fraction (%)	58 (48–64)	59 (46—64)	*0.66*
Aortic valve area (cm^2^)	0.8 (± 0.3)	0.8 (± 0.2)	*0.35*
Pmean (mmHg)	39 (± 15.5)	41 (± 15.7)	*0.17*
**CT**			
Bicuspid aortic valve (%)	82 (17%)	97 (18%)	*0.67*
Min. anulus diameter (mm)	21 (± 2.3)	21 (± 2.4)	*0.66*
Max. anulus diameter (mm)	27 (± 3)	27 (± 3.1)	*0.60*
**Laboratory parameters**			
Glomerular filtration rate (ml/min)	54 (± 20)	55 (± 20.4)	*0.49*
Platelet count (gpt/l)	219 (± 73.4)	223 (± 75.3)	*0.45*
NT-proBNP (ng/l)	1809 (713–4569)	1642 (724—4641)	*0.88*
**Antiplatelet therapy**			
Single antiplatelet therapy	199 (41%)	195 (36%)	*0.10*
Dual antiplatelet therapy	39 (8%)	32 (6%)	*0.18*
**Anticoagulation**			
Direct oral anticoagulants	160 (33%)	216 (40%)	***0.02***
Vitamin-K-antagonists	29 (6%)	22 (4%)	*0.16*

Values are mean (± SD) / median (IQR) depending on distribution or no (%)

CKD = Chronic kidney disease, PAD = peripheral artery disease, CAD = coronary artery disease, MI = myocardial infarction, CABG = coronary artery bypass grafting, SAVR = surgical aortic valve replacement, PCI = percutaneous coronary intervention, TAVI = transcatheter aortic valve implantation, TEER = transcatheter edge-to-edge repair, INR = International Normalized Ratio, NT-pro BNP = N-terminal prohormone of brain natriuretic peptide, RM = Angiographic roadmap, US = Ultrasound

* defined as a glomerular filtration rate < 60 ml/min/1.73 m2

### Procedural characteristics

Some significant differences in procedural characteristics were observed (Table 2). The sheath sizes and the sheath-to-femoral-artery ratio (SFAR) however, were evenly distributed among both groups. The use of large-bore plug-based vascular closure devices (MANTA®, Teleflex, Pennsylvania, USA) was significantly higher in the RM-group, whereas suture-based vascular closure devices (Proglide®, Abbott, Illinois, USA) were more common in the US group.

**Table 2:** Procedural characteristics

**Sheath size**	**RM (n = 485)**	**US (n = 541)**	*p-value*
14–16 FR	464 (96%)	521 (96%)	*0.61*
18–24 FR	21 (4%)	20 (4%)	*0.61*
**Anatomical features**			
Main access via right CFA	415 (86%)	465 (86%)	*0.86*
Severe calcification of the iliofemoral arteries	118 (24%)	88 (16%)	** < 0.001**
Severe tortuosity of the iliofemoral arteries	132 (27%)	62 (11%)	** < 0.001**
SFAR	0.66 ± 0.15	0.66 ± 0.15	*0.98*
**Procedural features**			
Procedure time (min)	52 (44–67)	49 (41–62)	**0.02**
Fluoroscopy time (min)	14 (11–20)	13 (10–17)	** < 0.001**
Contrast amount (ml)	88 (69–111)	84 (65–110)	**0.049**
**Vascular closure device**			
Suture-based vascular closure device	367 (76%)	521 (96%)	** < 0.001**
Plug-based vascular closure device	118 (24%)	20 (4%)	** < 0.001**
**Valve type**			
Self-expanding	276 (57%)	330 (61%)	*0.18*
Balloon-expandable	189 (39%)	200 (37%)	*0.51*

Values are mean (± SD) / median + (IQR) depending on distribution or no (%)

FR = French, CFA = Common femoral artery, SFAR = Sheath to femoral artery ratio, min = Minutes, ml = Milliliter

Patients in the RM-group presented more frequently with less favorable anatomy of the iliofemoral arteries due to more tortuosity and calcification as determined by the preprocedural CTA. Balloon-expandable and self-expanding valves were used similarly in both cohorts *(*Table 2*)*.

Significantly more contrast medium was used and fluoroscopy time was longer in the RM-group compared to the US group (contrast use: 88 [69–111] ml vs. 84 [65–110] ml, p = 0.049; fluoroscopy time: 14 [11–20] min vs. 13 [10–17] min, p < 0.001). Total procedure time (52 [44–67] min vs. 49 [41–62] min, p = 0.02) was significantly lower in the US-group *(*Table 2*, Central illustration)*.

### Primary and secondary clinical endpoints

**Fig. 1:**
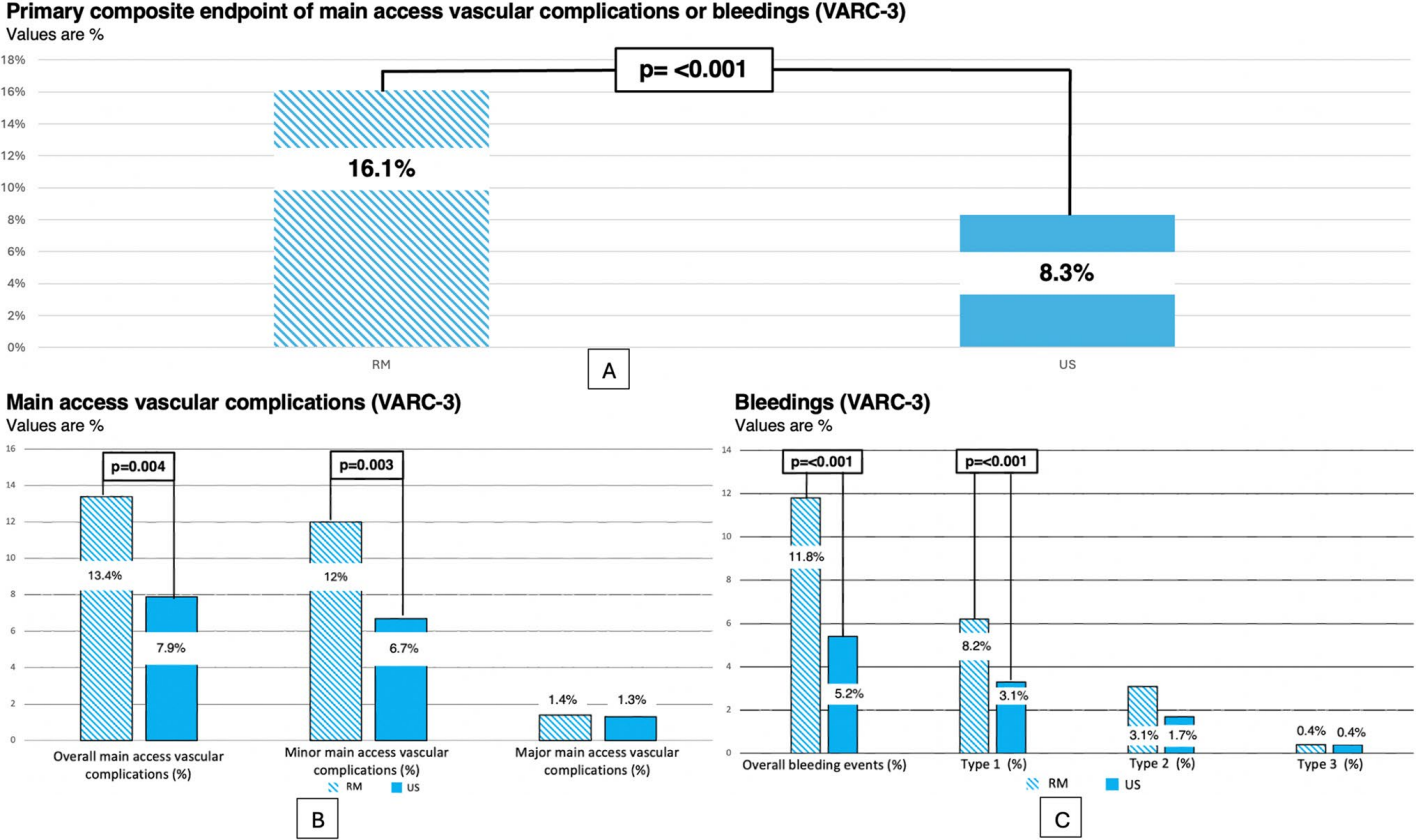
Primary endpoint and secondary endpoints. **A**: Rate of main access vascular complications or bleeding, defined by the Valve Academic Research Consortium 3 (VARC-3). **B**: Rate of vascular complications at the main access site, defined by the Valve Academic Research Consortium 3 (VARC-3). C: Rate of bleeding events, defined by the Valve Academic Research Consortium 3 (VARC-3). RM = Angiographic Roadmap; US = Ultrasound**.** Values are numbers (%)

The incidence of the primary composite endpoint was significantly higher in the RM-guided group compared to the US-guided group (16.1% [n = 78] vs. 8.3% [n = 45], p < 0.001) *(*Table 3*, *Fig. 1*, Central Illustration)*. This association remained statistically significant after multivariable adjustment (adjusted OR 0.51, 95% confidence interval [CI] 0.33–0.77, p = 0.002) *(*Table 4*)*.

**Table 3:** Procedural outcomes

**Primary endpoint**	**RM (n = 485)**	**US (n = 541)**	*p-value*
Main access vascular complications or bleedings	78 (16.1%)	45 (8.3%)	** < 0.001**
**Main access vascular complications**			
Total	65 (13.4%)	43 (7.9%)	**0.004**
- Minor	58 (12%)	36 (6.7%)	**0.003**
- Major	7 (1.4%)	7 (1.3%)	*0.84*
**Bleeding events**			
Total	57 (11.8%)	28 (5.2%)	** < 0.001**
- Type 1	40 (8.2%)	17 (3.1%)	** < 0.001**
- Type 2	15 (3.1%)	9 (1.7%)	*0.13*
- Type 3	2 (0.4%)	2 (0.4%)	*0.91*
- Type 4	-	-	-
**Accessory access vascular complications**			
Total	15 (3.1%)	9 (1.7%)	*0.13*
- Minor	10 (2.1%)	8 (1.5%)	*0.48*
- Major	5 (1%)	1 (0.2%)	*0.08*
**Acute kidney** **injury (VARC-3)**			
Total	58 (12%)	67 (12.4%)	*0.84*
- Stage 1	43 (8.9%)	48 (8.9%)	*0.99*
- Stage 2	7 (1.4%)	6 (1.1%)	*0.63*
- Stage 3	4 (0.8%)	6 (1.1%)	*0.64*
- Stage 4	4 (0.8%)	7 (1.3%)	*0.53*
**Mortality/Stroke**			
Mortality	3 (0.6%)	4 (0.7%)	*0.81*
Stroke	10 (2.1%)	14 (2.6%)	*0.58*

Values are no (%)

RM = Angiographic “Roadmap” guidance US = Ultrasound VARC-3 = Valve academic research consortium 3

**Table 4:** Multivariable analysis of the primary endpoint

**Predictors** Overall main access vascular complications or bleedings (VARC-3)	**Univariable**			**Multivariable**		
	**OR**	**95%-CI**	**p-value**	**Adjusted OR**	**95%-CI**	**p-value**
Age (years)	0.99	0.97–1.03	0.93	-	-	-
**Sex (f)**	**1.42**	**0.97–2.07**	**0.07**	1.35	0.90–2.01	0.15
BMI	0.99	0.96–1.03	0.81	-	-	-
**STS-Score**	**1.04**	**0.99–1.09**	**0.10**	1.04	0.98–1.09	0.21
**Hypertension**	**0.60**	**0.27–1.32**	**0.20**	0.60	0.26–1.37	0.22
Diabetes	1.12	0.77–1.65	0.55	-	-	-
PAD	1.08	0.56–2.10	0.81	-	-	-
Platelet count	1.00	0.99–1.00	0.76	-	-	-
VKA	1.12	0.50–2.55	0.78	-	-	-
DOAC	1.10	0.75–1.63	0.62	-	-	-
SAPT	0.96	0.65–1.41	0.82	-	-	-
DAPT	0.90	0.42–1.92	0.78	-	-	-
**US-guided-access**	**0.47**	**0.32–0.70**	** < 0.001**	**0.51**	**0.33–0.77**	**0.002**
Sheath-Size	0.99	0.96–1.01	0.79	-	-	-
**Suture-Based-VCD**	**0.51**	**0.30–0.86**	**0.01**	0.70	0.40–1.23	0.22
**SFAR**	**6.96**	**2.63–18.42**	** < 0.001**	**6.16**	**2.20–17.20**	**0.001**
**Severe iliofemoral calcification**	**1.62**	**1.05–2.48**	**0.03**	1.22	0.75–1.97	0.42
**Severe iliofemoral tortuosity**	**1.23**	**0.78–1.95**	**0.37**	1.02	0.62–1.67	0.94

Values are numbers

OR: odds ratio, CI: confidence interval

BMI = Body mass index, STS = Society of thoracic surgeons, PAD = Peripheral artery disease, VKA=Vitamin- K-Antagonist, DOAC = Direct oral anticoagulant, SAPT = Single antiplatelet therapy, DAPT = Dual antiplatelet therapy, US = Ultrasound, VCD = Vascular closure device, SFAR = Sheath-to-femoral-artery ratio

Vascular complications at the main access site were lower in the US-guided group (13.4% [n = 65] vs. 7.9% [n = 43], p = 0.02), with the difference persisting after adjustment for confounders (adjusted OR 0.59, 95% CI 0.38–0.92, p = 0.02) *(*Table 3* and Supplemental Table* S2*)*. This reduction was primarily driven by a lower rate of minor vascular complications at the main access site (RM-guided: 12% [n = 58] vs. US-guided: 6.7% [n = 36], p = 0.003), while major main access vascular complications were rare and occurred at comparable rates in both groups (RM-guided: 1.4% [n = 7] vs. US-guided: 1.3% [n = 7], p = 0.84) (Table 3*, *Fig. 1).

Accessory access site complications were rare, with a cumulative incidence of 3.1% (n = 15) in the RM-group versus 1.7% (n = 9) in the US group *(*Table 3*)*.

**Fig. 2:**
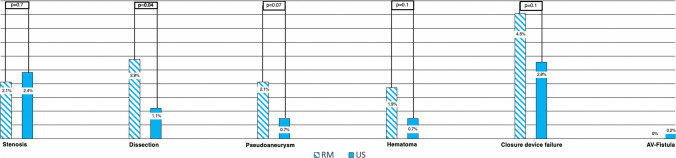
Type of vascular complications. Distribution of documented main access vascular complications. RM = Angiographic Roadmap; US = Ultrasound. Values are numbers (%)

The most common main access site vascular complications included closure device failure and stenosis of the iliofemoral arteries. There were significantly more dissections in the RM-group (2.9% [n = 14] vs. 1.1% [n = 6], p = 0.04) (*Supplemental Table* S1, Fig. 2).

A total of 57 (11.8%) bleeding events were observed in the RM-group compared with 28 events (5.2%) in the US group (p < 0.001, adjusted OR 0.46, 95% CI 0.28–0.78, p = 0.003) *(*Table 3* and Supplemental Table* S3*)*. Most of them were classified as type 1 (8.2% [n = 40] vs. 3.1% [n = 17], p = 0.001) and type 2 (3.1% [n = 15] vs. 1.7% [n = 9], p = 0.13) bleedings, while type 3 bleedings were rare and occurred equally in both groups (0.4% [n = 2] vs. 0.4% [n = 2], p = 0.91) (Table 3*, *Fig. 1).

**Fig. 3:**
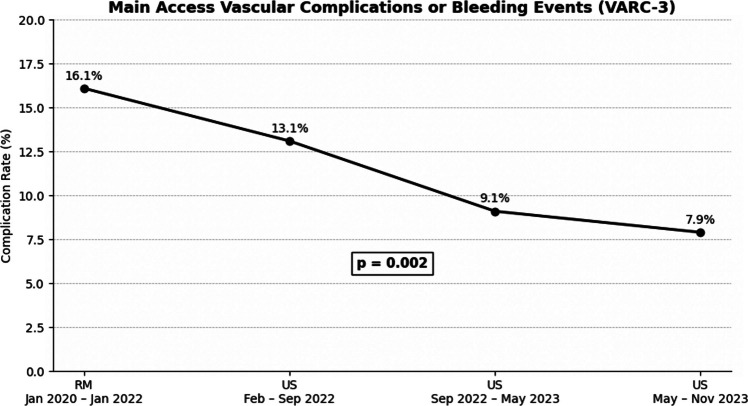
Learning curve. Rate of main access vascular complications or bleeding, defined by the Valve Academic Research Consortium 3 (VARC-3), in periods where RM was used (01/2020–02/2022), initial US phase (02/2022–09/2022), intermediate US phase (09/2022–04/2023) and advanced US phase (04/2023–11/2023). RM = Angiographic Roadmap; US = Ultrasound**.** Values are numbers (%)

We analyzed the occurrence of the primary endpoint during the RM-period and across three consecutive 7-month intervals following the introduction of US guidance to assess a potential learning curve: initial US-phase (02/2022–09/2022), intermediate US-phase (09/2022–04/2023), and advanced US-phase (04/2023–11/2023). During the initial US-phase, complications decreased modestly to 13.1% compared to 16.1% in the RM-phase. This downward trend continued in the intermediate and advanced US-phases, with complication rates dropping significantly to 9.1% and 7.9% (p = 0.002) respectively, indicating a progressive improvement over time (Fig. 3).

There were no significant differences in stroke (2.1% vs. 2.6%, p = 0.58) or in-hospital mortality (0.6% vs. 0.7%, p = 0.81) between both groups. Rates of acute kidney injury were also similar across both groups *(*Table 3*)*.

### Predictors for vascular complications and bleedings

US-guided access was identified as the only independent factor associated with a lower risk of the composite endpoint of main access vascular or bleeding complications (adjusted OR 0.51, 95% CI 0.33–0.77, p = 0.002) and overall main access vascular complications (adjusted OR 0.59, 95% CI 0.38–0.92, p = 0.02) by the multivariable analysis. *(*Table 4*, Supplemental Table* S2*)*.

A predictor for the occurrence of the composite primary endpoint or overall main access vascular complications was the SFAR, with an adjusted OR of 6.16 (95% CI 2.20–17.20, p = 0.001) and 6.61 (95% CI 2.3–19, p < 0.001), respectively *(*Table 4*, Supplemental Table* S2*).*

US guidance (OR 0.46, 95% CI 0.28–0.78, p = 0.003) and the use of a suture-based vascular closure device (OR 0.48, 95% CI 0.26–0.87, p = 0.016) were identified as protective predictors for the occurrence of bleeding events defined by VARC-3-criteria. The SFAR was a strong predictor of bleeding events (OR: 5.14, 95% CI:1.61–16.41, p = 0.006) *(Supplemental Table* S3*)*.

## Discussion

This study demonstrates that routine US-guidance for femoral arterial puncture in patients undergoing transfemoral TAVI is associated with a significant reduction in the risk of vascular or bleeding complications compared to RM-guidance. To the best of our knowledge, this is the first large-scale intra-operator comparison of US- and RM-guided femoral access for transfemoral TAVI, specifically focusing on high-volume operators with extensive expertise in RM-guided puncture who subsequently adopted an exclusively US-guided approach.

Transfemoral access is the preferred route for TAVI, accounting for over 90% of cases [19]. Traditionally, femoral puncture has relied on anatomic landmarks and fluoroscopic guidance with contralateral contrast injection (for example using the RM-technique). In contrast, US guidance provides real-time, cross-sectional visualization of vessel anatomy, potentially reducing vascular injuries and improving closure device performance. Although some society guidelines endorse US-guided access [20], there is still a lack of definitive evidence of its superiority over angiographic guidance during TAVI [14, 21].

In this context, we demonstrated that a change in practice to US-guided access for transfemoral TAVI is associated with a significant reduction in main access vascular and bleeding complications, as well as shorter procedure times and reduced contrast and fluoroscopy use compared to angiography-based guidance. Our findings also confirm and extend the results of the recent meta-analysis by Kotronias et al., which demonstrated substantial reductions in vascular and bleeding complications with US guidance [14]. These findings align with the recently published data from the PULSE registry, which, in a propensity score–matched analysis of 1790 patients undergoing transfemoral TAVI between 2016 and 2021 across multiple German centers, demonstrated a reduction in access-related vascular complications and type 2–4 bleeding events [15]. This supports a potential benefit of US-guided access in multicenter settings. However, the exclusive use of a single access technique by some centers may have introduced center-specific confounders, limiting the possibility of direct intra-operator comparisons. Our study addresses this limitation through an intra-operator design. Both operators at the same center transitioned from angiography-guided to US-guided access during the study period, effectively controlling for variability in operator skill and institutional protocols. This design allows for a more accurate assessment of the intrinsic benefits of US guidance, independent of external confounders. Moreover, the distinct change of the puncture technique towards US guidance at a clearly defined time point prevented the occurrence of selection bias. Our results underscore that even a seemingly small change, such as switching the primary access modality to US, can substantially enhance the safety and effectiveness of transfemoral TAVI. This is a crucial consideration for operators who currently rely on fluoroscopy or angiography-guided techniques. Moreover, we observed a pronounced learning curve associated with US-guided access. While this may present an initial challenge, it should not discourage operators from adopting this technique, given its demonstrated advantages.

The reduction in complications was mainly driven by fewer minor vascular events. Although less severe, these may still require further interventions (e.g., stenting, prolonged dual antiplatelet therapy), delay recovery, and compromise future transfemoral access, an important consideration for younger patients who may require further procedures in the future. US guidance also resulted in significantly fewer iliofemoral dissections, emphasizing its advantage in real-time vessel visualization and precise puncture control, particularly in avoiding anterior wall calcifications.

The lower bleeding rates in the US group likely reflect the combination of fewer vascular complications and the more frequent use of suture-based closure devices. The latter was identified as a protective factor in our multivariable analysis. Furthermore, the reduction in contrast and fluoroscopy use reflects improved procedural efficiency and may contribute to renal protection, although the differences were small and no differences in acute kidney injury were observed in our cohort.

This study has several limitations. First, it is a retrospective analysis of a single-center prospectively collected registry, with the inherent limitations of such a design. Although rigorous data collection was performed to mitigate this, the findings reflect real-world practice by highly experienced operators treating an unselected population. Second, as with all observational studies, the presence of unmeasured confounders cannot be fully excluded. Third, the sequential design may have introduced temporal bias favoring the US group. Fourth, operator experience, although already high, continued to increase during the study period and could have influenced outcomes. Fifth, the RM-group was more affected by the COVID-19 pandemic, potentially impacting procedural logistics, although clinical standards were largely maintained. Finally, these results reflect the practice of two highly experienced operators in a single high-volume center and may not be directly generalizable to lower-volume centers or operators with less experience in femoral access techniques.

## Conclusions

The current study provides robust real-world evidence supporting US-guidance as an effective strategy to reduce vascular and bleeding complications and to enhance procedural efficiency in transfemoral TAVI compared with an angiography-based approach, even among highly experienced operators. These findings strengthen the role of US-guidance as the preferred access strategy, particularly as TAVI continues to expand into younger and lower-risk populations. Randomized studies are warranted to confirm these results and to validate their generalizability across different centers and operator experience levels.

## Supplementary information

Below is the link to the electronic supplementary material.

ESM 1 (DOCX 23.7 KB)

## Abbreviations

CABG: Coronary artery bypass grafting
CAD: Coronary artery disease
CFA: Common femoral artery
PAD: Peripheral arterial disease
RM: Angiographic roadmap
SAVR: Surgical aortic valve replacement
TAVI: Transcatheter aortic valve implantation
TEER: Transcatheter edge-to-edge repair
US: Ultrasound
VARC-3: Valve Academic Research Consortium 3
SFAR: Sheath-to-femoral-artery ratio

## References

[1] Hayashida K, Lefvre T, Chevalier B et al (2011) Transfemoral Aortic Valve Implantation: New Criteria to Predict Vascular Complications. JACC Cardiovasc Interv 4(8):851–858. 10.1016/J.JCIN.2011.03.019 21851897 10.1016/j.jcin.2011.03.019

[2] Abdel-Wahab M, Hartung P, Dumpies O et al (2022) Comparison of a pure plug-based versus a primary suture-based vascular closure device strategy for transfemoral transcatheter aortic valve replacement: the choice-closure randomized clinical trial. Circulation 145(3):170–183. 10.1161/CIRCULATIONAHA.121.057856 34738828 10.1161/CIRCULATIONAHA.121.057856

[3] Mehilli J, Jochheim D, Abdel-Wahab M et al (2016) One-year outcomes with two suture-mediated closure devices to achieve access-site haemostasis following transfemoral transcatheter aortic valve implantation. EuroIntervention 12(10):1298–1304. 10.4244/EIJV12I10A213 27866140 10.4244/EIJV12I10A213

[4] Winter MP, Bartko P, Hofer F et al (2020) Evolution of outcome and complications in TAVR: a meta-analysis of observational and randomized studies. Sci Rep 10(1):15568. 10.1038/s41598-020-72453-1 32968104 10.1038/s41598-020-72453-1 PMC7511292

[5] Maniotis C, Andreou C, Karalis I, Koutouzi G, Agelaki M, Koutouzis M (2017) A systematic review on the safety of Prostar XL versus ProGlide after TAVR and EVAR. Cardiovasc Revasc Med 18(2):145–150. 10.1016/j.carrev.2016.11.004 27887905 10.1016/j.carrev.2016.11.004

[6] Blankenberg S, Seiffert M, Vonthein R et al (2024) Transcatheter or surgical treatment of aortic-valve stenosis. N Engl J Med 390(17):1572–1583. 10.1056/NEJMOA2400685 38588025 10.1056/NEJMoa2400685

[7] Mack MJ, Leon MB, Thourani VH et al (2023) Transcatheter aortic-valve replacement in low-risk patients at five years. N Engl J Med 389(21):1949–1960. 10.1056/NEJMOA2307447 37874020 10.1056/NEJMoa2307447

[8] Mack MJ, Leon MB, Thourani VH et al (2019) Transcatheter aortic-valve replacement with a balloon-expandable valve in low-risk patients. N Engl J Med 380(18):1695–1705. 10.1056/NEJMOA1814052 30883058 10.1056/NEJMoa1814052

[9] Martinsson A, Li X, Andersson C, Nilsson J, Smith JG, Sundquist K (2015) Temporal trends in the incidence and prognosis of aortic stenosis: a nationwide study of the Swedish population. Circulation 131(11):988–994. 10.1161/CIRCULATIONAHA.114.012906 25779541 10.1161/CIRCULATIONAHA.114.012906

[10] Bonow RO, Greenland P (2015) Population-wide trends in aortic stenosis incidence and outcomes. Circulation 131(11):969–971. 10.1161/CIRCULATIONAHA.115.014846 25691712 10.1161/CIRCULATIONAHA.115.014846

[11] Seto AH, Abu-Fadel MS, Sparling JM et al (2010) Real-time ultrasound guidance facilitates femoral arterial access and reduces vascular complications. JACC Cardiovasc Interv 3(7):751–758. 10.1016/j.jcin.2010.04.015 20650437 10.1016/j.jcin.2010.04.015

[12] Meijers TA, Nap A, Aminian A et al (2024) Ultrasound-guided versus fluoroscopy-guided large-bore femoral access in PCI of complex coronary lesions: the international, multicentre, randomised ULTRACOLOR trial. EuroIntervention 20(14):e876-e886. 10.4244/EIJ-D-24-00089 38742577 10.4244/EIJ-D-24-00089 PMC11228538

[13] Iannopollo G, Nobile G, Lanzilotti V et al (2022) Percutaneous artErial closure devices and ultrasound-guided trans-femoRal puncture ObservatioNal InvestigatiOn: insights from the PETRONIO registry. Catheter Cardiovasc Interv 99(3):795–803. 10.1002/CCD.29828 34137485 10.1002/ccd.29828

[14] Kotronias RA, Bray JJH, Rajasundaram S et al (2021) Ultrasound- versus fluoroscopy-guided strategy for transfemoral transcatheter aortic valve replacement access: a systematic review and meta-analysis. Circ Cardiovasc Interv. 10.1161/CIRCINTERVENTIONS.121.010742 34538068 10.1161/CIRCINTERVENTIONS.121.010742 PMC8522629

[15] Grundmann D, Rudolph T, Adam M et al (2025) Procedural and clinical outcomes according to ultrasound-guided access in TAVI: a propensity-matched comparative subanalysis from the PULSE registry. Circ Cardiovasc Interv. Published online. https://doi.org/10.1161/CIRCINTERVENTIONS.124.014771/ASSET/E87A8CE2-3F05-4EB8-9FD7-A979F2363D00/ASSETS/GRAPHIC/CIRCINTERVENTIONS.124.014771.ABSTRACT1.JPG. Accessed 22 July 2025 10.1161/CIRCINTERVENTIONS.124.014771 40438925

[16] Rheude T, Ruge H, Altaner N et al (2025) Comparison of strategies for vascular ACCESS closure after transcatheter aortic valve implantation: the ACCESS-TAVI randomized trial. Eur Heart J 46(7):635–645. 10.1093/EURHEARTJ/EHAE784 39474906 10.1093/eurheartj/ehae784

[17] Grundmann D, Kim W, Kellner C et al (2025) A propensity-matched comparison of plug- versus suture-based vascular closure after TAVI. EuroIntervention 21(5):e272-e281. 10.4244/EIJ-D-24-00120 40028729 10.4244/EIJ-D-24-00120 PMC11849536

[18] Généreux P, Piazza N, Alu MC et al (2021) Valve Academic Research Consortium 3: updated endpoint definitions for aortic valve clinical research. Eur Heart J 42(19):1825–1857. 10.1093/eurheartj/ehaa799 33871579 10.1093/eurheartj/ehaa799

[19] Meertens MM, Adam M, Beckmann A et al (2025) Non-femoral focused transaxillary access in TAVI: GARY data analysis and future trends. Clin Res Cardiol 114(3):323–331. 10.1007/S00392-024-02402-9/TABLES/3 38436739 10.1007/s00392-024-02402-9 PMC11913932

[20] Naidu SS, Aronow HD, Box LC et al (2016) SCAI expert consensus statement: 2016 best practices in the cardiac catheterization laboratory: (endorsed by the cardiological society of India, and sociedad Latino Americana de Cardiologia intervencionista; affirmation of value by the Canadian Association of interventional cardiology–Association canadienne de cardiologie d’intervention)*. Catheter Cardiovasc Interv 88(3):407–423. 10.1002/CCD.26551 27137680 10.1002/ccd.26551

[21] Kotronias RA, Scarsini R, De Maria GL et al (2020) Ultrasound guided vascular access site management and left ventricular pacing are associated with improved outcomes in contemporary transcatheter aortic valve replacement: insights from the OxTAVI registry. Catheter Cardiovasc Interv 96(2):432–439. 10.1002/ccd.28578 31742885 10.1002/ccd.28578

