# Supplementary material for: Ultrasound versus angiographic guided access in transfemoral TAVI: intra-operator evaluation of vascular and bleeding complications
Source: Clin Res Cardiol. 2025 Dec 15;115(7):1230–40. doi: 10.1007/s00392-025-02805-2 (PMC13249720; doi:10.1007/s00392-025-02805-2)
Supplement: Supplementary file 1 — (DOCX 23.7 KB) [file 392_2025_2805_MOESM1_ESM.docx]

**Supplement:**

**Table 1** – Types of main access site related vascular complication

| **Types of main access vascular complications** | **RM (n=485)** | **US (n=541)** | *p-value* |
| --- | --- | --- | --- |
| Stenosis | 10 (2.1%) | 13 (2.4%) | *0.71* |
| Dissection | 14 (2.9%) | 6 (1.1%) | **0.04** |
| Pseudoaneurysm | 10 (2.1%) | 4 (0.7%) | *0.07* |
| Hematoma | 9 (1.9%) | 4 (0.7%) | *0.11* |
| Vascular closure device failure | 22 (4.5%) | 15 (2.8%) | *0.13* |
| AV-Fistula | 0 (0%) | 1 (0.2%) | -- |
| Values are no (%).  RM = Angiographic “Roadmap” guidance US = Ultrasound | | | |

**Table 2** – Multivariate analysis of overall main access vascular complications

| **Predictors**  Overall main access vascular complications (VARC-3) | **Univariate** | | | **Multivariate** | | |
| --- | --- | --- | --- | --- | --- | --- |
|  | **OR** | **95%-CI** | **p-value** | **Adjusted OR** | **95%-CI** | **p-value** |
| Age (yrs) | 0.99 | 0.96 – 1.03 | 0.76 | - | - | - |
| **Sex (f)** | **1.26** | **0.84 – 1.88** | **0.26** | 1.21 | 0.79 – 1.84 | 0.38 |
| BMI | 0.99 | 0.96 – 1.04 | 0.93 | - | - | - |
| **STS-Score** | **1.04** | **0.99 – 1.09** | **0.18** | 1.03 | 0.97 – 1.08 | 0.37 |
| **Hypertension** | **0.61** | **0.26 – 1.40** | **0.24** | 0.59 | 0.25 – 1.41 | 0.24 |
| Diabetes | 1.05 | 0.70 – 1.60 | 0.80 | - | - | - |
| PAD | 1.30 | 0.57 – 2.26 | 0.73 | - | - | - |
| Platelet count | 1.00 | 0.99 – 1.00 | 0.89 | - | - | - |
| VKA | 1.09 | 0.46 – 2.61 | 0.85 | - | - | - |
| DOAC | 1.04 | 0.69 – 1.57 | 0.86 | - | - | - |
| SAPT | 1.07 | 0.71 – 1.61 | 0.73 | - | - | - |
| DAPT | 0.75 | 0.32 – 1.77 | 0.51 | - | - | - |
| **US-guided-access** | **0.56** | **0.37 – 0.84** | **0.005** | **0.59** | **0.38 – 0.92** | **0.02** |
| Sheath-Size | 0.99 | 0.99 – 1.01 | 0.81 | - | - | - |
| **Suture-Based-VCD** | **0.6** | **0.33 – 1.02** | **0.06** | 0.76 | 0.41 – 1.39 | 0.37 |
| **SFAR** | **7.69** | **2.81 – 21.06** | **<0.001** | **6.61** | **2.30 – 18.97** | **<0.001** |
| **Severe iliofemoral calcification** | **1.7** | **1.01 – 2.67** | **0.02** | 1.29 | 0.79 – 2.10 | 0.30 |
| Severe iliofemoral tortuosity | 1.1 | 0.67 – 1.82 | 0.69 |  |  |  |
| Values are numbers.  OR: odds ratio, CI: confidence interval.  BMI = Body mass index, STS = Society of thoracic surgeons, PAD = Peripheral artery disease, VKA=Vitamin-K-Antagonist, DOAC = Direct oral anticoagulant, SAPT = Single antiplatetelet therapy, DAPT = Dual antiplatelet therapy, US = Ultrasound, VCD = Vascular closure device, SFAR = Sheath-to-femoral-artery ratio | | | | | | |

**Table 3** – Multivariate analysis of overall bleedings

| **Predictors**  Overall bleeding events (VARC-3) | **Univariate** | | | **Multivariate** | | |
| --- | --- | --- | --- | --- | --- | --- |
|  | **OR** | **95%-CI** | **p-value** | **adjusted OR** | **95%-CI** | **p-value** |
| **Age (yrs)** | **1.02** | **0.98 – 1.06** | **0.36** | 1.01 | 0.97 – 1.05 | 0.56 |
| **Sex (f)** | **1.26** | **0.81 – 1.97** | **0.31** | 1.12 | 0.70 – 1.79 | 0.64 |
| BMI | 1.01 | 0.97 – 1.05 | 0.67 | - | - | - |
| **STS-Score** | **1.05** | **1.00 – 1.11** | **0.049** | 1.05 | 0.99 – 1.12 | 0.09 |
| **Hypertension** | **0.69** | **0.27 – 1.80** | **0.45** | 0.79 | 0.29 – 2.15 | 0.64 |
| Diabetes | 1.09 | 0.69 – 1.70 | 0.72 | - | - | - |
| PAD | 0.82 | 0.35 – 1.93 | 0.65 | - | - | - |
| Platelet count | 1.00 | 0.99 – 1.00 | 0.93 | - | - | - |
| VKA | 1.16 | 0.45 – 3.00 | 0.76 | - | - | - |
| DOAC | 1.47 | 0.94 – 2.30 | 0.09 | - | - | - |
| SAPT | 0.97 | 0.61 – 1.53 | 0.90 | - | - | - |
| DAPT | 0.99 | 0.42 – 2.36 | 0.98 | - | - | - |
| **US-guided-access** | **0.41** | **0.26 – 0.66** | **<0.001** | **0.46** | **0.28 – 0.78** | **0.003** |
| Sheath-Size | 0.99 | 0.99 -1.01 | 0.88 | - | - | - |
| **Suture-Based-VCD** | **0.36** | **0.21 – 0.63** | **<0.001** | **0.48** | **0.26 – 0.87** | **0.016** |
| **SFAR** | **5.52** | **1.89 – 16.14** | **0.002** | **5.14** | **1.61 – 16.41** | **0.006** |
| **Severe iliofemoral calcification** | **1.53** | **0.92 – 2.54** | **0.01** | 1.12 | 0.64 – 1.97 | 0.70 |
| **Severe iliofemoral tortuosity** | **1.26** | **0.73 – 2.15** | **0.41** | 1.01 | 0.57 – 1.80 | 0.98 |
| Values are numbers.  OR: odds ratio, CI: confidence interval.  BMI = Body mass index, STS = Society of thoracic surgeons, PAD = Peripheral artery disease, VKA=Vitamin-K-Antagonist, DOAC = Direct oral anticoagulant, SAPT = Single antiplatetelet therapy, DAPT = Dual antiplatelet therapy, US = Ultrasound, VCD = Vascular closure device, SFAR = Sheath-to-femoral-artery ratio | | | | | | |
